# Idiopathic pulmonary fibrosis: diagnostic pitfalls and therapeutic challenges

**DOI:** 10.1186/2049-6958-7-42

**Published:** 2012-11-12

**Authors:** Paolo Spagnolo, Roberto Tonelli, Elisabetta Cocconcelli, Alessandro Stefani, Luca Richeldi

**Affiliations:** 1 Center for Rare Lung Disease, University Hospital of Modena, Via del Pozzo 71, 41124, Modena, Italy

**Keywords:** Clinical trials, Idiopathic pulmonary fibrosis, Interstitial lung disease, Pulmonary fibrosis, Treatment

## Abstract

Idiopathic pulmonary fibrosis (IPF), the most common of the idiopathic interstitial pneumonias, is a devastating condition that carries a prognosis worse than that of many cancers. As such, it represents one of the most challenging diseases for chest physicians. The diagnostic process is complex and relies on the clinician integrating clinical, laboratory, radiologic, and/or pathologic data. Therefore, a close collaboration between chest physicians, radiologists, and pathologists experienced in the diagnosis of interstitial lung diseases (ILDs) is necessary in order to minimize diagnostic uncertainty. Similarly, the management of IPF continues to pose major difficulties. However, while there are no proven effective therapies for IPF beyond lung transplantation, recent trials of novel agents suggest that pharmacological treatment may retard the progression of the disease. In this regard, enrolment of patients into clinical trials is considered the “best current practice”by the most recent guidelines as it offers IPF patients the chance to receive new agents that may be more effective than current therapies. A more recent trend focusing on improving quality of life in IPF patients has also been gaining ground.

The diagnosis and management of IPF remains a constant challenge for even the most experienced of clinicians. However, a multidisciplinary approach to this complex disease is steadily improving diagnostic accuracy, while recent advances in the pharmacological therapy offer the genuine promise of future treatments for this devastating disease.

## Introduction

Idiopathic pulmonary fibrosis (IPF) is a progressive fibrosing interstitial pneumonia of unknown cause, limited to the lung and associated with the histopathological (evidence of patchy involvement of lung parenchyma by fibrosis/architectural distortion, honeycombing in a predominantly subpleural/paraseptal distribution, presence of fibroblast foci) and/or radiological (subpleural, basal-predominant honeycombing and reticular abnormality, with or without traction bronchiectasis) pattern of usual interstitial pneumonia (UIP) [1]. The disease carries a dismal prognosis with a median survival time - in retrospective longitudinal studies - of 2 to 3 years from the time of diagnosis [2-5]. The diagnosis of IPF is established in the presence of a UIP pattern on high-resolution computed tomography (HRCT) of the chest and/or in the surgical lung biopsy (SLB) specimen in the appropriate clinical setting (commonly a current or ex-smoker male of >60 years of age) and after the exclusion of all known causes of pulmonary fibrosis [1]. However, the diagnosis may not be straightforward; in fact, IPF belongs to a large family which is estimated being populated by more than 200 different entities, known as interstitial lung diseases (ILDs), many of which have features similar to IPF [6]. One recent study estimated the prevalence of all ILDs in the US at about 500,000 [7]. Not surprisingly, >50% of these patients are initially misdiagnosed with other forms of respiratory illness [8].

Diagnosing IPF in daily practice may be challenging and there is often a significant delay between the first manifestations of the disease - typically a combination of dyspnea on exertion and dry cough - and its correct diagnosis [9]. Moreover, a sufficient level of diagnostic confidence is not reached in a sizeable proportion of cases. Disease management is similarly challenging. To date, only one drug has been approved for use in Europe, India and Japan and there are no licensed medical therapies in the US: more importantly, no medical therapy has proven to improve overall survival in patients with IPF [10]. As such, the only care options that are endorsed by the recent evidence-based guidelines are lung transplantation and enrolment in a clinical trial [1]. However, less than one-third of a large patient cohort evaluated at a center with extensive expertise in ILD could realize these currently recommended treatment options, highlighting the need for other management approaches [11].

This review focuses on current diagnostic and therapeutic challenges in IPF. Timely diagnosis and early referral of patients to centers with specific expertise may lead to more optimal disease management.

## Diagnostic challenges

### Delay in diagnosis

Suspicion of IPF often arises several months after the initial manifestations of the disease. In fact, dry cough and exertional dyspnea - the most common presenting symptoms - tend to be overlooked and wrongly attributed to smoking habits or aging. Uncertainties, thus diagnostic delay, may be due to a number of reasons. Sometimes, patients may be too sick to undergo surgical, although relatively non-invasive, procedures and this may preclude obtaining tissue samples, which are critical for the final diagnosis in a sizeable proportion of cases. Conversely, in community centers the diagnostic delay could be due to the radiologist or pathologist insufficient experience with ILD. Nevertheless, although the overall accuracy of a diagnosis of IPF in expert centers has been reported to be good (87%), even in this setting the level of agreement within experts is fair to moderate [12]. Significant disagreement between academic and community-based physicians with regard to the final diagnosis also exists, with community-based physicians being more likely to assign a final diagnosis of IPF compared with academic physicians, highlighting the importance of referring patients with suspected IPF to centers with expertise in ILD [13].

### Ruling out alternative diagnoses

Being the disease “idiopathic” by definition, the diagnosis of IPF requires the exclusion of all known causes of ILD. Because the number of potential “mimickers” of IPF is high, this step is crucial.

#### Familial pulmonary fibrosis

Familial pulmonary fibrosis (FPF), the occurrence of IPF in two or more members of the same family, accounts for less than 5% of total IPF cases [14,15]. Familial and sporadic IPF are clinically and histologically indistinguishable, although familial forms may develop at an earlier age [14] and display different patterns of gene transcription [16]. Cigarette smoking appears to be a risk factor for the development of FPF, suggesting that environmental/occupational exposures may accentuate genetic risk and that gene-environment interactions may be critical in IPF pathogenesis. Another remarkable finding is the pathologic heterogeneity within family members. In fact, while 50% of the families have a “uniform” diagnosis of UIP/IPF, the remaining 50% display radiological or pathologic features suggestive of a different IIP in at least one affected family member [17].

#### Hypersensitivity pneumonitis

Patients with ILD should be thoroughly investigated for possible HP since chronic forms - commonly caused by prolonged exposure to low antigen levels - may mimic IPF both clinically and radiologically [18]. Bronchoalveolar lavage (BAL) showing a lymphocytosis of 40% or greater, mainly sustained by CD8+ T lymphocytes, strongly suggests the diagnosis of chronic HP [19]. Search for possible exposures should cover occupational and non-occupational causes of HP with particular attention to domestic mold and fungal and bacterial agents. If diagnostic uncertainty persists the diagnosis requires histologic confirmation, although chronic forms of HP may display a UIP pattern. Nonetheless, recognition of the causal antigen is critical not only for the diagnosis but also for the treatment as avoidance of further exposure is essential [20].

#### Connective tissue disease

Connective tissue disease (CTD) can present with a UIP pattern [21] and ILD has been described as the sole clinical manifestation of some of these conditions [22,23]. Because IPF is rare before the age of 50, the index of suspicion for an underlying CTD should be high in the presence of young patients, especially female, since patients without clinical or serologic features at presentation may subsequently manifest overt features of an underlying CTD that was subclinical at the time of first presentation. Conversely, patients with IPF may have positive antinuclear antibodies (ANA) at low titers and/or borderline positive rheumatoid factor without any other clinical features of CTD [24]. Such patients should be carefully screened for signs and symptoms of connective tissue disease, e.g., arthritis, Raynaud’s phenomenon, skin changes or abnormal esophageal motility. If repeat serologic and clinical evaluation during follow up confirms the development of a CTD, the diagnosis should be revised, given the therapeutic and prognostic implications [1].

#### Asbestosis

The differential diagnosis between asbestosis and IPF may not be straightforward owing to the radiological and histopathological similarities between these two entities [25]. Since the aetiology, behaviour and prognosis of these two conditions may differ significantly, an accurate differentiation is strictly needed, also considering that patients with asbestosis may be eligible for legal compensation [26]. Chest HRCT features of asbestos-induced pulmonary fibrosis and IPF may overlap in some cases and look similar at first sight [27]. Emphasis must be placed on the presence of pleural involvement (e.g., pleural plaques or diffuse pleural thickening), which is almost universal in asbestosis [26]. However, relying on pleural disease alone may be an oversimplification: in fact, an individual previously exposed to asbestos remains as susceptible to non-asbestos-induced fibrotic lung diseases (including IPF) as the general population; on the other hand, diffuse pleural thickening may be due to other causes [28]. Histologically, some cases of asbestosis resemble UIP - while others resemble fibrotic nonspecific interstitial pneumonitis (NSIP) - although the presence of asbestos bodies may permit to differentiate asbestosis from other ILDs [27]. In addition, fibroblastic foci, a pathologic hallmark of IPF, are infrequent in asbestosis [27]. Finally, asbestosis is almost invariably characterized by mild fibrosis of the visceral pleura, a rare feature in IPF. If uncertainty persists, specific fibre analysis may be necessary to determine the aetiology of the fibrotic process [27].

#### Sarcoidosis

Without an adequate review of a patient’s medical history and past relevant imaging data, fibrotic sarcoidosis (radiographic stage IV; [29,30]) may be overlooked. HRCT features of pulmonary sarcoidosis include upper lobe-predominant small nodules distributed along the broncho-vascular bundle and pleural membranes, thickening of the interlobular septa and mediastinal lymphadenopathy. However, subpleural bibasal honeycombing, cysts and traction bronchiectasis mimicking a UIP pattern may rarely be observed [31-33]. Findings in support of a diagnosis of sarcoidosis include a history of skin or ocular lesions, hilar or mediastinal lymphadenopathy, chronic fatigue, and elevated serum levels of angiotensin-converting enzyme (ACE) [34-36]. Conversely, chest HRCT appearances compatible with sarcoidosis may prove to be atypical manifestations of IPF [37].

Table 1 lists the main radiologic and histopathologic features that help differentiate IPF from other fibrotic lung diseases.

**Table 1:** Radiologic and histopathologic features suggestive of a fibrotic lung disease other than IPF

	**Hypersensitivity pneumonitis**	**Connective tissue disease**	**Asbestosis**	**Sarcoidosis**
**Radiologic features**	Areas of decreased attenuation	Bronchiectasis far from fibrotic areas	Plaques and/or significant pleural thickening	Fibrosis, linear opacities and traction bronchiectasis predominantly in the perihilar regions and upper lobes
	Centrilobular nodules Mid-upper lobe predominance	Signs of pulmonary hypertension disproportionate to the extent of fibrosis	Limited extent	Conglomerate masses of fibrosis in the posterior part of the lungs
		Pleural or pericardial effusion Oesophagus or bone abnormalities		Small well-defined nodules with a perilymphatic distribution
**Histological features**	Cellular interstitial pneumonia	Dense perivascular collagen	Pleural abnormalities	Non-caseating granulomas with a characteristic perilymphatic distribution
	Multinucleated giant cells or granulomas situated around bronchioles	Extensive pleuritis	Asbestos bodies	
		Lymphoid aggregates with germinal center formation	Bronchial wall fibrosis	
		Prominent plasmacytic infiltration	Fibroblastic foci infrequent	

### Special conditions

#### IPF presenting as acute exacerbation of IPF

The clinical course of IPF is most commonly characterized by a slowly progressive decline in lung function and worsening dyspnea leading to death within several years of diagnosis [38]. However, IPF may also present, though rarely, acutely in patients without a known history of chronic lung disease [39]. In such cases, lung biopsy (often difficult to obtain due to patient clinical conditions) shows diffuse alveolar damage (DAD) superimposed on a UIP pattern, similar to what is seen in acute exacerbations of IPF (AE-IPF). AE-IPF is defined as an acute unexplained respiratory deterioration (worsening dyspnea, increased cough and worsening of gas exchange parameters) within one month and the appearance of new parenchymal opacities on chest radiograph or HRCT in the absence of alternate causes, such as infection, pulmonary embolism or heart failure [40]. The correct diagnosis may remain uncertain if the patient conditions contraindicate a confirmatory lung biopsy; as such, these patients are often misdiagnosed as acute interstitial pneumonia or acute respiratory distress syndrome.

#### Unclassifiable ILD

In some cases, even at the end of an accurate diagnostic work-up, the nature of the fibrotic process may remain unclear. Possible causes of diagnostic failure could be either technical or atypical manifestations of the disease. For instance, a biopsy taken from the “wrong” area may provide normal lung tissue, indiscernible end-stage lung or a sample that does not meet diagnostic criteria for any specific ILD. In such cases, the biopsy will not be diagnostic.

### Challenges in obtaining a surgical lung biopsy

In patients whose HRCT does not demonstrate a UIP pattern, a surgical lung biopsy (SLB) is necessary to make a definitive diagnosis. From a technical point of view, the following aspects should be considered: type of surgical approach, side of operation, target areas for the biopsy and number of biopsies. The diagnostic yield from surgical lung biopsies obtained from video-assisted thoracoscopy (VATS) and open thoracotomy are similar, although VATS is associated with lower morbidity and length of stay than open thoracotomy, thus representing the procedure of choice [1], while a lateral “muscle sparing” thoracotomy should be limited to cases with strong pleural adhesions, which makes VATS not practicable. Target areas for biopsy should be selected on the basis of HRCT features and discussed with the radiologist. Ideally, biopsy specimens should be taken neither from the most fibrotic areas nor from normal areas. In patients with suspected IPF, biopsies should be obtained from multiple lobes. In fact, specimens obtained from different segments may display discordant histological patterns, although cases with coexisting UIP pattern and fibrotic NSIP pattern (*discordant UIP*) appear to behave similarly to those with UIP pattern in all lobes (*concordant UIP*) [41,42]. If both lungs are equally affected, SLB is commonly performed in the left lung, because lung margins are thinner and easy to resect; in addition, right lung is usually functionally dominant, thus deflating the left lung may allow the patient to better tolerate single-lung ventilation. After SLB, a morbidity of 9-20% and a mortality of 0-5% have been reported, this disagreement likely reflecting the different types of patients included in the SLB series [43]. The decision whether or not to obtain a SLB must be tailored to the clinical situation of the individual patient and after a careful evaluation of benefits and risks. Thus, while in younger patients (potential candidates to lung transplantation) an histological confirmation of the diagnosis is strongly recommended, in older patients with severe physiologic impairment or substantial comorbidity, the risks of SLB may outweigh the benefits of establishing a secure diagnosis of IPF.

Table 2 summarizes the main challenges in diagnosing IPF.

**Table 2:** Diagnostic challenges in IPF

**Delay in diagnosis**
**Ruling out alternative diagnoses**
Familial pulmonary fibrosis
Hypersensitivity pneumonitis
Connective Tissue Disease
Asbestosis
Sarcoidosis
**Special conditions**
IPF presenting as acute exacerbation of IPF
Unclassifiable ILD
**Obtaining a surgical lung biopsy**

## Therapeutic challenges

### Pharmacological therapies

#### Improving survival

IPF is a disease with a poor prognosis that appears unchanged over the past decade: the median survival in non-transplanted patients is around 3.5 years after diagnosis [1,11]. Therefore, at present, improving survival appears the most difficult goal to achieve [44]. In fact, none of the studies that aimed at showing an effect on survival either as primary or secondary endpoint showed evidence of efficacy in this respect [45]. This could be due to a number of factors, including disease heterogeneity, concomitant conditions influencing clinical response, incomplete knowledge of disease pathogenetic mechanisms, real lack of efficacy of the compounds tested thus far, need to tackle more than one fibrogenic pathway, which in turn, would require a multi-drug regimen, or case selection, since clinical trials have often included highly selected patients while many are excluded by virtue of their age, disease severity, or comorbid conditions. In this regard, it is noteworthy that of IPF patients evaluated at a referral US centre over a period of 5 years less than one third qualified for enrolment in a clinical trial, despite the availability and contiguity of multiple clinical trials [11]. Conversely, treatments initially thought to slow the decline of lung function [46] or reduce mortality in patients experiencing acute exacerbation [47] and which received a *weak recommendation* against their use by the most recent guidelines [1] have subsequently been shown not only to be ineffective but even harmful in patients with IPF [48,49]. Specifically, an efficacy and safety interim analysis of the PANTHER-IPF (Prednisone, Azathioprine, and N-acetylcysteine: A Study That Evaluates Response in IPF) study, in which patients with mild-to-moderate lung function impairment were randomized in a 1:1:1 ratio to prednisone, azathioprine and NAC (combination therapy), NAC alone or placebo, revealed that the combination therapy, as compared to placebo, was associated with a statistically significant increase in all-cause mortality (11% vs. 1%), all-cause hospitalizations (29% vs. 8%), and treatment-related severe adverse events (31% vs. 9%) [49], thus providing robust evidence against the use of this combination of drugs in patients with IPF.

There is a growing appreciation that distinct phenotypes exist within what is currently regarded as IPF. Clinical variants include a temporal spectrum of disease behavior that ranges from a rapid, including acute exacerbations, to a slowly protracted pattern of progression. Interestingly, this latter “survivor” group is equally distributed among disease severity groups [11]. A survival “tail” has been described in older publications but it has been commonly attributed to the inclusion of patients with diseases other than IPF (i.e., NSIP); having excluded alternative diagnoses, the observed survival “tail” in IPF appears to be real [11]. These patients should not be included in clinical trials assessing survival as *slow progressors* are less likely to respond to any given drug, thus leading to false negative results.

#### Improvement of clinically meaningful outcomes

Drugs evaluated to date in IPF have failed to demonstrate an effect on survival [1]. However, recent data suggest that treatment may impact on a number of clinically meaningful outcomes. Forced vital capacity (FVC) is the most commonly used physiological measurement and primary endpoint in clinical trials in IPF. In fact, changes in a patient’s FVC over time (whether analyzed continuously or categorically as above or below a threshold value) have been correlated with survival time in multiple large cohorts of patients with IPF [1,50]. Recently, in a 12-month, phase 2 trial, BIBF 1120 - an intracellular tyrosine kinase inhibitor - has been shown to reduce by 68% the annual rate of decline in FVC as compared with placebo in patients with IPF (0.06 liters vs. 0.19 liters) [51]. Similarly, BIBF 1120 was associated with improved health-related quality of life and reduced frequency of acute exacerbations, which, in turn, are associated with rapid disease progression, severe and abrupt decline in FVC, and high mortality.

### Treatment of concomitant conditions

There is an increasing awareness of complications and comorbidities frequently associated with IPF [1] but it is unknown whether they are related to the underlying pathobiology of IPF or whether they simply reflect concurrent diseases of aging. Likewise, it is unclear whether IPF patients should be screened for these conditions or whether treatment of concomitant conditions affects disease outcome, although, their presence influences both the eligibility of patients for lung transplants and their survival while on the waiting list. In addition, comorbidities and the medications prescribed for them can often contribute to symptoms, thus hampering diagnosis and treatment [52].

Common concomitant conditions include pulmonary hypertension (PH), vascular or coronary artery disease, gastroesophageal reflux disease, obesity, diabetes, emphysema, and obstructive sleep apnoea [1]. PH is common in IPF and is associated with lower diffusing capacity of carbon monoxide (DL_CO_), shorter walk distance, desaturation during exercise and increased risk of death [38]. In a retrospective study of IPF patients undergoing pre-transplantation right heart catheterization, one-year mortality rates were higher in patients with PH (28.0% vs. 5.5% amongst patients without PH) - with mortality risk linearly correlating with mean pulmonary artery pressure [53]. Higher pulmonary vascular resistance and PH are also likely to account for the poor prognosis observed in patients with combined pulmonary fibrosis and emphysema [54]. In addition, recent evidence suggests that PH is associated with the development of acute exacerbations and, in turn, with poor prognosis [55]. Whether targeting of PH with medications approved for the treatment of pulmonary arterial hypertension (PAH) has any utility in the context of IPF remains unclear. In a small open label trial sildenafil - an oral phosphodiesterase 5 inhibitor - improved 6-minute-walk distance and pulmonary hemodynamics without increasing shunt flow or worsening oxygenation [56]. However, a subsequent large multicenter, randomized, double-blind, placebo-controlled study did not meet the primary end-point (change of 20% in 6-minute-walk distance [6MWD] at 12 weeks), although statistically significant differences were observed in dyspnea, PaO_2_, DL_CO_, and quality of life favouring sildenafil [57]. Bosentan - an endothelin (ET)_A_ and ET_B_ receptor antagonist - has also been studied in IPF. Based on a non-predefined subset analysis of a smaller study suggesting that bosentan may prevent disease progression in patients that had undergone a diagnostic surgical lung biopsy [58], a larger prospective, double-blind, placebo-controlled trial was conducted. Unfortunately, the primary endpoint (time to IPF worsening or death up to end of the study) was not met [59]. Similarly, a randomized, double-blind, placebo-controlled trial of ambrisentan - a selective antagonist of the (ET)_A_ receptor - has been prematurely stopped following an interim analysis indicating lack of efficacy (http://www.gilead.com/pr*,* December 22 2010).

Gastroesophageal reflux disease (GERD) is another common comorbidity in patients with IPF (prevalence of approximately 90%), and the likelihood of having both IPF and GERD increases in patients >60 years of age [60]. Patients with IPF and GERD are at higher risk for hospitalizations from any cause as well as from respiratory illness, thus suggesting that recurrent epithelial injury caused by microaspiration of gastric juice and contents may causes alveolar damage and pulmonary fibrosis [61]. The prevalence of microaspiration in patients with IPF is not known, and it is not clear whether microaspiration represents an intrinsic risk factor or causes acute exacerbations of IPF. However, in a recent uncontrolled retrospective study the reported use of GERD medications was associated with longer survival in patients with IPF, supporting the hypothesis that GERD and chronic microaspiration may play important roles in the pathobiology of IPF [62].

Coronary artery disease (CAD) is more prevalent in IPF patients compared to a similarly matched chronic obstructive pulmonary disease (COPD) group; this association, which appears to be independent of common CAD risk factors, carries a worse prognosis [63,64].

At present there are no solid, prospective data on which to make definitive recommendations for treatment of concomitant conditions in patients with IPF.

### Management of acute exacerbations of IPF

Acute exacerbations of IPF (AE-IPF) are, by definition, idiopathic. Therefore, known causes of acute deterioration, such as pulmonary embolism, congestive heart failure, pneumothorax and infection need to be thoroughly searched for and excluded. Based on data derived from recent randomized clinical trials, the yearly incidence of AE-IPF is between 10 and 15% of all patients [51,65,66]. The prognosis of AE-IPF is poor, with mortality ranging from 78% to 96% [67]. Although current guidelines recommend the use of high-dose corticosteroids, pharmacological treatment of AE-IPF is largely empiric and usually consists of intravenous corticosteroids up to a gram per day, variably combined with immunosuppressive drugs. At present, there are no controlled clinical trials on which to judge efficacy of this therapeutic strategy and specific recommendations regarding dosage, route and duration of therapy cannot be made. Many of the patients experiencing acute deterioration require intensive care treatment, particularly when respiratory failure is associated with hemodynamic instability, significant co-morbidities or severe hypoxemia. However, mortality during hospitalization is high [68]. In addition, patients with end-stage interstitial lung disease are difficult to ventilate and rarely successfully weaned from mechanical ventilation [69-71]. Thus, while the decision not to ventilate an IPF patient with acute respiratory failure is a tricky one, mechanical ventilation should be introduced only after carefully weighing the patient’s long-term prognosis and, whenever possible, the patient’s wishes. However, current guidelines discourage the use of mechanical ventilation in patients with respiratory failure secondary to IPF [1].

## Non-pharmacological interventions

### Timely referral to a tertiary care center

IPF is often initially misdiagnosed - at least until physiological and imaging data suggest the presence of an interstitial lung disease - leading to delay in accessing appropriate care. This point is not trivial in a disease with a median survival of 3 years after diagnosis. In a prospective cohort study of 129 IPF patients Lamas and coworkers showed that delayed access to a tertiary care center (defined as time from the onset of dyspnea to the date of initial evaluation at a tertiary care center) is associated with a higher risk of death independent of disease severity [72]. In turn, a delay in receiving a correct diagnosis of IPF might lead to initiation of ineffective or harmful interventions [49] and may delay evaluation for lung transplantation and inclusion in clinical trials [73]. Early referral to a center with specific expertise should therefore be considered for any patient with suspected or known interstitial lung disease.

### Lung transplantation

Lung transplant (LTx) represents the only treatment of proven benefit in IPF. In fact, in IPF patients lung transplant has been shown to reduce the risk of death by 75% as compared with patients who remain on the waiting list [74]. Since the introduction of the Lung Allocation Score (LAS), which prioritizes transplant candidates based on survival probability, IPF has become the most common indication for LTx in the United States. High LAS values are associated with decreased survival following lung transplantation, increased length of stay following transplantation, and higher rates of infection, renal failure and stroke [75]. Symptomatic patients with IPF younger than 65 years should be referred for lung transplantation if there is a DL_CO_ < 39% predicted and/or evidence of a FVC decline > 10% over 6 months [76], but there are no clear data to guide the precise timing for LTx. Although controversial, the most recent data suggest that bilateral LTx is superior to single LTx in patients with IPF [77].

### Pulmonary rehabilitation

Lack of energy and fatigue are common and disabling problems for patients with IPF. Pulmonary rehabilitation (PR) may alleviate symptoms and improve functional status by stabilizing and/or reversing the extra-pulmonary features of the disease [78,79]. Typical PR programs include exercise training, nutritional modulation, occupational therapy, education and psychosocial counseling. Previous studies, in which physical training was compared to no physical training or other therapy, were not limited to patients with IPF, and included conditions potentially more amenable to the beneficial effect of PR. In a randomized-controlled trial on the effect of exercise-based PR in patients with ILD, including 34 with IPF, Holland and colleagues observed that the increase in 6MWD and the reduction in dyspnea and fatigue among IPF patients were not as remarkable as among the non-IPF patients. In addition, these benefits were seen immediately following training but not sustained 6 months after intervention [78]. With time IPF patients tend to discontinue any routine exercise due to increasing dyspnea. Whenever possible, this should be discouraged.

### Palliative care

Although the key goal of treatment in IPF is to prevent disease progression, palliating symptoms such as dyspnea, chronic cough, depression and anxiety is equally important [80]. In addition, because the goal of maintaining a maximum level of wellness and quality of life requires the active patient participation, education about the disease and its course is another central component of care in IPF management. In this regard, psychosocial support through counseling or patient support groups is critical in addressing unique needs of patients with IPF.

In advanced disease dyspnea can be extremely distressing, thus impairing physical activity and quality of life. In selected cases of particularly severe dyspnea morphine could be considered. In a small case series Allen and colleagues reported that low dose diamorphine reduces dyspnea, anxiety and cough without significant decrease in oxygen saturation [81]. Further, oxygen therapy may be useful for palliation of dyspnea in hypoxemic patients. With disease progression patients may experience fear, anxiety and depression; psychological counseling and, in selected cases, pharmacological treatment should therefore be considered. In a recent cross-sectional study of outpatients with interstitial lung disease, including IPF, Ryerson and colleagues reported that depression score, functional status (as assessed by 4 minutes walk test) as well as pulmonary function all contribute to the severity of dyspnea [82]. Of note, this study shows that the relationship between dyspnea and depression is independent of other clinical variables, suggesting that treating depression (observed in as many as 23% of patients in this study) and functional status may improve dyspnea and quality of life.

Therapeutic challenges in IPF are summarized in Table 3.

**Table 3:** Therapeutic challenges in IPF

**Pharmacologic treatment**
Improving survival
Improvement of clinically meaningful outcomes
Treatment of concomitant conditions
Management of acute exacerbations of IPF
**Non pharmacologic treatment**
Timely referral to a tertiary care center
Lung transplantation
Pulmonary rehabilitation
Palliative care

## Conclusions

The diagnosis and management of IPF represent a difficult task even for the most experienced clinicians. The disease should always be suspected in patients aged ≥50 years current or ex-smokers complaining of dry cough and dyspnea, particularly in the presence of compatible HRCT features [83]. However, the diagnosis may not be straightforward: HRCT features are typical (“definite” according to current guidelines) in only half of the cases, while biopsy-proven diagnoses are available in a minority of patients, even though histopathologic information has the greatest impact on the final diagnosis, particularly when the initial clinical/radiographic diagnosis is not IPF [6]. Early diagnosis would offer benefits to patients in terms of adequate information on the disease, timely referral for lung transplantation, avoidance of inappropriate drugs (e.g., steroids and immunosuppressive) [49], and enrollment in clinical trials. However, the symptoms of early IPF are often subtle, while ILD screening efforts are limited to subjects with known risk factors or with a history of familial IPF. How can we reduce diagnostic uncertainty? Recognition or suspicion (e.g., unexplained dyspnea) of IPF should prompt early referral to a specialty center. In fact, the diagnosis of IPF requires close collaboration between clinicians, radiologists, and pathologists experienced in ILD, and this is particularly relevant in cases in which the radiologic and histopathologic patterns are discordant (e.g., HRCT features *inconsistent* with UIP and histopathology showing UIP). Multidisciplinary specialist clinics and coordinated services can be only found in dedicated centers with high-volume ILD programs. Referral to centers with expertise may also allow participation in a clinical trial.

Management of IPF is similarly challenging. The only care options that are endorsed by current guidelines are lung transplantation and enrolment in a clinical trial [1]. In the past decade better understanding of the patho-biology of the disease has led to a dramatic increase in the number of therapies evaluated in IPF. Although participation in a clinical trial is not without risk, including side-effects from the drug under investigation, this option offers the patients the opportunity to play an active role in their own healthcare, gain access to new, potentially beneficial treatments as well as obtaining expert medical care at leading healthcare facilities [80]. In addition, clinical trials have provided crucial information about the natural history of the disease, and may guide subsequent study designs.

The goals of any disease management strategy include improving quality of life, helping patients to be more functional, reducing disease-related morbidity and complications or helping people to live longer. Given the absence of a proven, effective therapy a large degree of therapeutic nihilism has always surrounded the management of IPF. However, a more recent trend focusing on improving symptoms and quality of life in IPF patients has been gaining ground, and it is now widely accepted that palliative care should be a treatment priority and an integral and routine component of patients care [80].

Much work remains to be done in order to detect IPF in preclinical and early stages; nevertheless, the diagnostic accuracy has significantly increased by means of multidisciplinary discussion between clinicians, radiologists and pathologists with expertise in ILD. Similarly, and perhaps more importantly, recent clinical trials have shown promise in identifying treatment options. It is hoped that the results from ongoing and new clinical trials will soon determine an effective treatment for patients with this devastating disease.

## Abbreviations

ACE: Angiotensin-converting enzyme; AE: Acute exacerbations; ANA: Antinuclear antibodies; BAL: Bronchoalveolar lavage; CAD: Coronary artery disease; COPD: Chronic obstructive pulmonary disease; CTD: Connective tissue disease; DL_co_: Diffusing capacity of carbon monoxide; ET: Endothelin; FPF: Familial pulmonary fibrosis; FVC: Forced vital capacity; GERD: Gastroesophageal reflux disease; HP: Hypersensitivity pneumonitis; HRCT: High-resolution computed tomography; ILDs: Interstitial lung diseases; IPF: Idiopathic pulmonary fibrosis; LAS: Lung Allocation Score; LTx: Lung transplant; NAC: N-acetylcysteine; NSIP: Nonspecific interstitial pneumonitis; PAH: Pulmonary arterial hypertension; PH: Pulmonary hypertension; PR: Pulmonary rehabilitation; 6MWD: 6-minute-walk distance; UIP: Usual interstitial pneumonia; VATS: Video-assisted thoracoscopy.

## Competing interests

LR reports receiving consulting fees from BoehringerIngelheim, Intermune, Celgene, Sanofi, Anthera and Gilead; lecture fees from Intermune. PS, RT, EC and AS have no conflict of interest to declare.

## Authors’ contributions

PS and LR conceived of the study and drafted the manuscript. RT, EC and AS participated in the design of the review and helped to draft the manuscript. All authors read and approved the final manuscript.
